# Penta­aqua­(di­methyl­formamide)­cobalt(II) sulfate di­methyl­formamide monosolvate

**DOI:** 10.1107/S1600536813012841

**Published:** 2013-06-08

**Authors:** Murat Taş, Seval Çamur, Sevim Topal

**Affiliations:** a Giresun University, Department of Chemistry, Art and Sciences Faculty, Giresun, Turkey

## Abstract

The title compound, [Co(C_3_H_7_NO)(H_2_O)_5_]SO_4_·C_3_H_7_NO, contains five aqua ligands, a Co^II^ atom, a sulfate ion and both a coordinating and a non-coordinating di­methyl­formamide (DMF) mol­ecule. The DMF solvent mol­ecule lies between the complex units, which are located along the *b* axis. The sulfate ion is for charge balance. The Co^II^ atom has distorted octa­hedral coordination geometry, being ligated by five aqua ligands and the O atom of the DMF ligand. O—H⋯O hydrogen bonds between the aqua ligands and the sulfate anion and non-coordinating DMF molecule lead to the formation of a three-dimensional network. Since all constituents lie on a mirror plane, the H atoms of all methyl groups and of one of the aqua ligands are equally disordered over two positions.

## Related literature

For background to the use of DMF, see: Kolthoff *et al.* (1970 ▶); Pastoriza-Santos & Liz-Marzan (1999 ▶); Kimmerle & Eben (1975 ▶); Gescher (1993 ▶); Zhou *et al.* (1996 ▶); Matwiyoff (1966 ▶). For amide complexes, see: Rao *et al.* (1984 ▶); Angus *et al.* (1993 ▶); Khum & Maclntyre (1965 ▶).
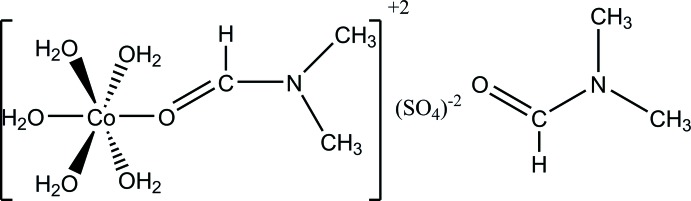


## Experimental

### 

#### Crystal data

[Co(C_3_H_7_NO)(H_2_O)_5_]SO_4_·C_3_H_7_NO
*M* *_r_* = 391.26
Orthorhombic, 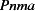

*a* = 22.256 (8) Å
*b* = 7.449 (7) Å
*c* = 9.929 (9) Å
*V* = 1646 (2) Å^3^
*Z* = 4
Mo *K*α radiation
μ = 1.22 mm^−1^
*T* = 298 K
0.28 × 0.20 × 0.19 mm

#### Data collection

Agilent SuperNova diffractometer
Absorption correction: multi-scan (SCALE3 in *ABSPACK*; Agilent, 2011 ▶) *T*_min_ = 0.956, *T*_max_ = 1.000
3903 measured reflections
1627 independent reflections
1407 reflections with *I* > 2σ(*I*)
*R*_int_ = 0.021

#### Refinement

*R*[*F* ^2^ > 2σ(*F* ^2^)] = 0.041
*wR*(*F* ^2^) = 0.100
*S* = 1.14
1627 reflections
139 parameters
3 restraints
H atoms treated by a mixture of independent and constrained refinement
Δρ_max_ = 0.65 e Å^−3^
Δρ_min_ = −0.38 e Å^−3^

### 

Data collection: *CrysAlis PRO* (Agilent, 2011 ▶); cell refinement: *CrysAlis PRO*; data reduction: *CrysAlis PRO*; program(s) used to solve structure: *SHELXS97* (Sheldrick, 2008 ▶); program(s) used to refine structure: *SHELXL97* (Sheldrick, 2008 ▶); molecular graphics: *OLEX2* (Dolomanov *et al.*, 2009 ▶); software used to prepare material for publication: *OLEX2*.

## Supplementary Material

Crystal structure: contains datablock(s) global, I. DOI: 10.1107/S1600536813012841/bq2385sup1.cif

Structure factors: contains datablock(s) I. DOI: 10.1107/S1600536813012841/bq2385Isup2.hkl

Additional supplementary materials:  crystallographic information; 3D view; checkCIF report

## Figures and Tables

**Table 1:** Selected bond lengths (Å)

Co1—O1	2.062 (4)
Co1—O2	2.101 (10)
Co1—O4	2.046 (4)
Co1—O3	2.110 (9)

**Table 2:** Hydrogen-bond geometry (Å, °)

*D*—H⋯*A*	*D*—H	H⋯*A*	*D*⋯*A*	*D*—H⋯*A*
O1—H1*A*⋯O6^i^	0.86	1.95	2.792 (4)	171
O1—H1*B*⋯O6^ii^	0.86	2.26	2.792 (4)	120
O3—H3*A*⋯O5^iii^	0.85 (2)	1.99 (2)	2.807 (8)	162 (4)
O3—H3*B*⋯O8^iv^	0.88 (2)	1.85 (2)	2.731 (17)	178 (4)
O2—H2*A*⋯O6^v^	0.88 (2)	1.79 (2)	2.660 (3)	171 (4)
O2—H2*B*⋯O7^iv^	0.89 (4)	1.86 (4)	2.745 (15)	177 (4)

Symmetry codes: (i) 
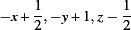
; (ii) 
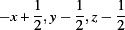
; (iii) 
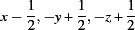
; (iv) 
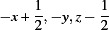
; (v) 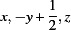.

**Fig. 1.**
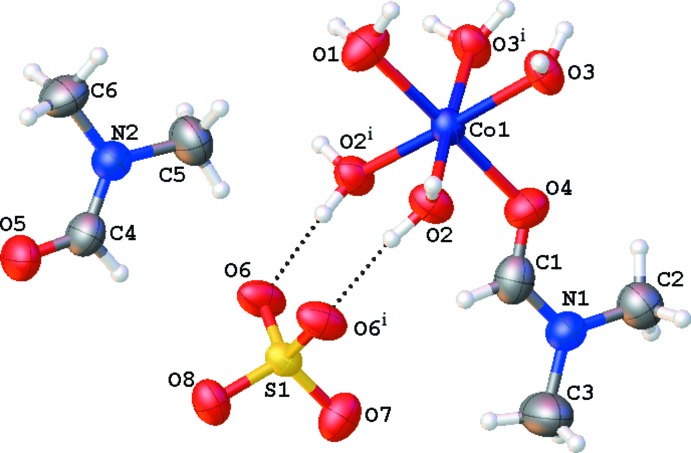
A view of the structure of the title complex, showing the atom labelling. i: x, 1/2-y, z

**Fig. 2.**
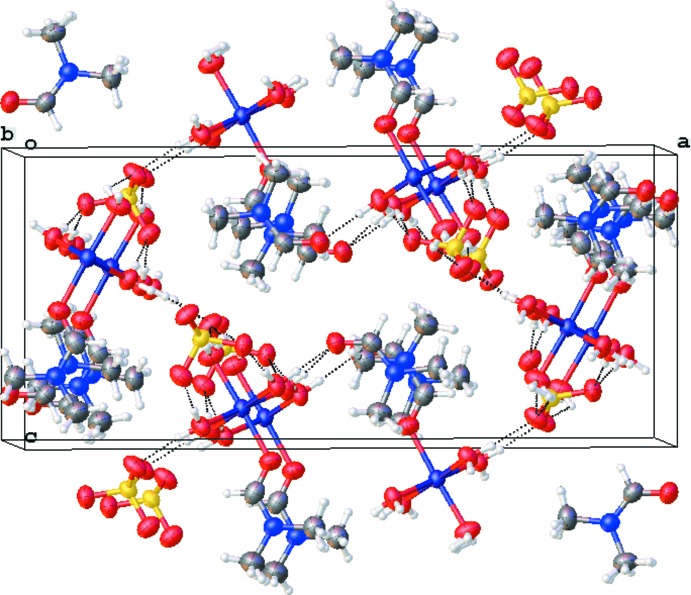
A view of the packing diagram of the titled compound.

## supplementary crystallographic information

### Comment

*N*,*N*-Dimethylformamide (DMF) which is a simple model molecule for the peptide bond in proteins and readily absorbed into the human organism by inhalitaion or dermal contamination and is suspected of being a carcinogen, is an important compound used as a solvent in a variety of industrial processes including the preparation of synthetic fibers, leathers, films, and surface coatings, preparation of colloids (Kolthoff *et al.*, 1970; Pastoriza-Santos & Liz-Marzan, 1999; Kimmerle & Eben, 1975; Gescher, 1993; Zhou *et al.*, 1996). Also DMF shows similar solvent properties to those of water and methanol and shows promise as a nonaqueous medium for ionic reactions (Matwiyoff, 1966).

Due to the model properties for peptides, the amide complexes is of continuing interest. Crystallographic studies have shown that in complexes, the amides are bonded to the metal atom by using their carbonyl oxygen (Rao *et al.*, 1984; Angus *et al.*, 1993; Khum & Maclntyre, 1965).

The asymmetric unit of the titled compound contains two different DMF molecules. One of them is acted as ligand and bonds to the Co(II) ions *via* its oxygen atom and the other one is involved as solvate molecules in the crystal structure. The structure also has sulfate ion to charge balance.

The Co(II) atom has distorted octahedral geometry, being ligated by five aqua ligands and a DMF ligand (Table 1). The coordination bond lengths were found 2.046 (7) Å for Co—O_DMF_ and in the rage of 2.062–2.110 Å for Co—O_aqua_. The O—H···O intermolecular hydrogen bonds formed three dimensional molecular network, in solid state. The sulphate ions plays major role to form the three-dimensional structure via formation of the hydrogen bonds. The DMF solvate units were capsulated between the complex units which locate along the *b*-axis, by the hydrogen bond interactions (Table 2).

### Experimental

The CoSO_4_.6H_2_O and 5-hydantoin acetic acid was mixed in 50 ml DMF solvent. The pH of the solution was adjusted to 6.7 by 1% NaHCO_3_ solution. The mixture was heated to 50°C and stirred for 1 h and then slowly cooled to room temperature. The solution was kept for several weeks, so suitable crystals for X-ray analyses was obtained.

### Refinement

Refinement of F^2^ against ALL reflections. The weighted *R*-factor *wR* and goodness of fit S are based on F^2^, conventional *R*-factors *R* are based on F, with F set to zero for negative F^2^. The threshold expression of F^2^ > 2σ(F^2^) is used only for calculating *R*-factors(gt) *etc*. and is not relevant to the choice of reflections for refinement. *R*-factors based on F^2^ are statistically about twice as large as those based on F, and R– factors based on ALL data will be even larger.

### Crystal data

**Table:**

[Co(C_3_H_7_NO)(H_2_O)_5_]SO_4_·C_3_H_7_NO	*D*_x_ = 1.578 Mg m^−^^3^
*M**_r_* = 391.26	Mo *K*α radiation, λ = 0.7107 Å
Orthorhombic, *P**n**m**a*	Cell parameters from 108 reflections
*a* = 22.256 (8) Å	θ = 4.4–25.9°
*b* = 7.449 (7) Å	µ = 1.22 mm^−^^1^
*c* = 9.929 (9) Å	*T* = 298 K
*V* = 1646 (2) Å^3^	Block, clear red
*Z* = 4	0.28 × 0.20 × 0.19 mm
*F*(000) = 820	

### Data collection

**Table:**

Agilent SuperNova (Single source at offset, Eos) diffractometer	1627 independent reflections
Radiation source: SuperNova (Mo) X-ray Source	1407 reflections with *I* > 2σ(*I*)
Mirror monochromator	*R*_int_ = 0.021
Detector resolution: 16.0454 pixels mm^-1^	θ_max_ = 25.6°, θ_min_ = 3.3°
ω scans	*h* = −27→24
Absorption correction: multi-scan (SCALE3 in ABSPACK; Agilent, 2011)	*k* = −5→8
*T*_min_ = 0.956, *T*_max_ = 1.000	*l* = −11→7
3903 measured reflections	

### Refinement

**Table:**

Refinement on *F*^2^	Primary atom site location: structure-invariant direct methods
Least-squares matrix: full	Secondary atom site location: difference Fourier map
*R*[*F*^2^ > 2σ(*F*^2^)] = 0.041	Hydrogen site location: inferred from neighbouring sites
*wR*(*F*^2^) = 0.100	H atoms treated by a mixture of independent and constrained refinement
*S* = 1.14	*w* = 1/[σ^2^(*F*_o_^2^) + (0.0358*P*)^2^ + 1.4684*P*] where *P* = (*F*_o_^2^ + 2*F*_c_^2^)/3
1627 reflections	(Δ/σ)_max_ < 0.001
139 parameters	Δρ_max_ = 0.65 e Å^−^^3^
3 restraints	Δρ_min_ = −0.38 e Å^−^^3^

### Special details

**Table:**

Geometry. All esds (except the esd in the dihedral angle between two l.s. planes) are estimated using the full covariance matrix. The cell esds are taken into account individually in the estimation of esds in distances, angles and torsion angles; correlations between esds in cell parameters are only used when they are defined by crystal symmetry. An approximate (isotropic) treatment of cell esds is used for estimating esds involving l.s. planes.
Refinement. Refinement of F^2^ against ALL reflections. The weighted R-factor wR and goodness of fit S are based on F^2^, conventional R-factors R are based on F, with F set to zero for negative F^2^. The threshold expression of F^2^ > 2sigma(F^2^) is used only for calculating R-factors(gt) etc. and is not relevant to the choice of reflections for refinement. R-factors based on F^2^ are statistically about twice as large as those based on F, and R- factors based on ALL data will be even larger.

### Fractional atomic coordinates and isotropic or equivalent isotropic displacement parameters (Å^2^)

**Table:**

	*x*	*y*	*z*	*U*_iso_*/*U*_eq_	Occ. (<1)
Co1	0.14060 (2)	0.2500	0.38601 (6)	0.0369 (2)	
O1	0.18765 (17)	0.2500	0.2071 (4)	0.0595 (10)	
H1A	0.1918	0.3576	0.1787	0.089*	0.50
H1B	0.1684	0.1889	0.1484	0.089*	0.50
O2	0.19603 (11)	0.0408 (3)	0.4542 (3)	0.0480 (6)	
O4	0.09678 (16)	0.2500	0.5671 (4)	0.0554 (9)	
O3	0.08065 (10)	0.0530 (3)	0.3135 (3)	0.0491 (6)	
N1	0.09188 (18)	0.2500	0.7912 (4)	0.0507 (11)	
C1	0.1212 (3)	0.2500	0.6752 (6)	0.0581 (14)	
H1	0.1630	0.2500	0.6774	0.070*	
C3	0.1242 (3)	0.2500	0.9139 (6)	0.090 (2)	
H3C	0.1223	0.1329	0.9538	0.135*	0.50
H3D	0.1069	0.3363	0.9743	0.135*	0.50
H3E	0.1654	0.2809	0.8968	0.135*	0.50
C2	0.0295 (2)	0.2500	0.7952 (6)	0.0671 (16)	
H2C	0.0154	0.1316	0.8164	0.101*	0.50
H2D	0.0140	0.2859	0.7091	0.101*	0.50
H2E	0.0160	0.3325	0.8630	0.101*	0.50
S1	0.31484 (5)	0.2500	0.66245 (12)	0.0364 (3)	
O8	0.37947 (14)	0.2500	0.6875 (4)	0.0512 (9)	
O7	0.28365 (16)	0.2500	0.7907 (4)	0.0557 (9)	
N2	0.40622 (18)	0.2500	0.2342 (4)	0.0489 (10)	
O5	0.49626 (15)	0.2500	0.3358 (4)	0.0607 (10)	
C6	0.4297 (3)	0.2500	0.0993 (5)	0.0689 (17)	
H6A	0.4029	0.1851	0.0413	0.103*	0.50
H6B	0.4685	0.1936	0.0987	0.103*	0.50
H6C	0.4334	0.3713	0.0680	0.103*	0.50
C4	0.4415 (2)	0.2500	0.3370 (6)	0.0546 (13)	
H4	0.4232	0.2500	0.4212	0.066*	
C5	0.3412 (2)	0.2500	0.2504 (7)	0.0722 (18)	
H5A	0.3313	0.2272	0.3430	0.108*	0.50
H5B	0.3239	0.1581	0.1948	0.108*	0.50
H5C	0.3254	0.3647	0.2243	0.108*	0.50
O6	0.29865 (11)	0.4135 (3)	0.5871 (2)	0.0539 (7)	
H3A	0.0516 (12)	0.090 (5)	0.266 (3)	0.067 (13)*	
H3B	0.0944 (16)	−0.044 (4)	0.274 (4)	0.067 (13)*	
H2A	0.2308 (11)	0.065 (6)	0.492 (4)	0.082 (15)*	
H2B	0.2032 (17)	−0.055 (6)	0.404 (4)	0.071 (14)*	

### Atomic displacement parameters (Å^2^)

**Table:**

	*U*^11^	*U*^22^	*U*^33^	*U*^12^	*U*^13^	*U*^23^
Co1	0.0354 (3)	0.0320 (4)	0.0434 (4)	0.000	−0.0010 (3)	0.000
O1	0.086 (3)	0.041 (2)	0.052 (2)	0.000	0.0169 (19)	0.000
O2	0.0492 (14)	0.0394 (14)	0.0553 (15)	0.0061 (12)	−0.0120 (12)	−0.0035 (13)
O4	0.063 (2)	0.059 (2)	0.044 (2)	0.000	0.0044 (17)	0.000
O3	0.0401 (13)	0.0398 (14)	0.0672 (17)	−0.0011 (11)	−0.0094 (12)	−0.0070 (13)
N1	0.051 (2)	0.057 (3)	0.044 (2)	0.000	0.0028 (19)	0.000
C1	0.054 (3)	0.052 (3)	0.068 (4)	0.000	0.011 (3)	0.000
C3	0.076 (4)	0.134 (7)	0.060 (4)	0.000	−0.007 (3)	0.000
C2	0.060 (3)	0.073 (4)	0.068 (4)	0.000	−0.003 (3)	0.000
S1	0.0398 (6)	0.0284 (6)	0.0410 (6)	0.000	−0.0073 (5)	0.000
O8	0.0391 (17)	0.046 (2)	0.069 (2)	0.000	−0.0041 (16)	0.000
O7	0.066 (2)	0.041 (2)	0.060 (2)	0.000	0.0174 (18)	0.000
N2	0.048 (2)	0.053 (3)	0.046 (2)	0.000	0.0012 (19)	0.000
O5	0.045 (2)	0.077 (3)	0.061 (2)	0.000	0.0062 (17)	0.000
C6	0.077 (4)	0.084 (4)	0.045 (3)	0.000	0.006 (3)	0.000
C4	0.051 (3)	0.061 (4)	0.052 (3)	0.000	0.010 (2)	0.000
C5	0.047 (3)	0.091 (5)	0.078 (4)	0.000	−0.003 (3)	0.000
O6	0.0689 (15)	0.0350 (13)	0.0578 (15)	−0.0014 (12)	−0.0243 (12)	0.0065 (12)

### Geometric parameters (Å, º)

**Table:**

Co1—O1	2.062 (4)	C3—H3E	0.9600
Co1—O2^i^	2.101 (10)	C2—H2C	0.9600
Co1—O2	2.101 (10)	C2—H2D	0.9600
Co1—O4	2.046 (4)	C2—H2E	0.9600
Co1—O3	2.110 (9)	S1—O8	1.460 (3)
Co1—O3^i^	2.110 (9)	S1—O7	1.450 (4)
O1—H1A	0.8552	S1—O6	1.474 (3)
O1—H1B	0.8552	S1—O6^i^	1.474 (3)
O2—H2A	0.878 (19)	N2—C6	1.437 (6)
O2—H2B	0.89 (4)	N2—C4	1.288 (7)
O4—C1	1.203 (7)	N2—C5	1.457 (6)
O3—H3A	0.850 (18)	O5—C4	1.219 (6)
O3—H3B	0.879 (19)	C6—H6A	0.9600
N1—C1	1.324 (7)	C6—H6B	0.9600
N1—C3	1.414 (7)	C6—H6C	0.9600
N1—C2	1.389 (6)	C4—H4	0.9300
C1—H1	0.9300	C5—H5A	0.9600
C3—H3C	0.9600	C5—H5B	0.9600
C3—H3D	0.9600	C5—H5C	0.9600
			
O1—Co1—O2	88.81 (11)	O4—C1—N1	123.6 (5)
O1—Co1—O2^i^	88.81 (11)	O4—C1—H1	118.2
O1—Co1—O3^i^	91.57 (12)	N1—C1—H1	118.2
O1—Co1—O3	91.57 (12)	N1—C3—H3C	109.5
O2—Co1—O2^i^	95.9 (5)	N1—C3—H3D	109.5
O2^i^—Co1—O3	176.13 (10)	N1—C3—H3E	109.5
O2^i^—Co1—O3^i^	88.0 (5)	N1—C2—H2C	109.5
O2—Co1—O3	88.0 (5)	N1—C2—H2D	109.5
O2—Co1—O3^i^	176.13 (10)	N1—C2—H2E	109.5
O4—Co1—O1	177.94 (15)	O8—S1—O6	109.10 (17)
O4—Co1—O2	89.81 (11)	O8—S1—O6^i^	109.10 (17)
O4—Co1—O2^i^	89.81 (11)	O7—S1—O8	108.8 (2)
O4—Co1—O3	89.90 (11)	O7—S1—O6	109.19 (18)
O4—Co1—O3^i^	89.91 (11)	O7—S1—O6^i^	109.19 (18)
O3^i^—Co1—O3	88.2 (5)	O6^i^—S1—O6	111.4 (5)
Co1—O1—H1A	109.8	C6—N2—C5	117.7 (5)
Co1—O1—H1B	109.5	C4—N2—C6	121.1 (5)
H1A—O1—H1B	109.1	C4—N2—C5	121.2 (5)
Co1—O2—H2A	120 (3)	N2—C6—H6A	109.5
Co1—O2—H2B	122 (3)	N2—C6—H6B	109.5
H2A—O2—H2B	104 (4)	N2—C6—H6C	109.5
C1—O4—Co1	124.7 (4)	N2—C4—H4	116.5
Co1—O3—H3A	116 (3)	O5—C4—N2	127.0 (5)
Co1—O3—H3B	120 (2)	O5—C4—H4	116.5
H3A—O3—H3B	106 (4)	N2—C5—H5A	109.5
C1—N1—C3	119.9 (5)	N2—C5—H5B	109.5
C1—N1—C2	121.2 (5)	N2—C5—H5C	109.5
C2—N1—C3	119.0 (5)		
			
Co1—O4—C1—N1	180.0	C3—N1—C1—O4	180.0
O2^i^—Co1—O4—C1	−47.92 (7)	C2—N1—C1—O4	0.000 (1)
O2—Co1—O4—C1	47.92 (7)	C6—N2—C4—O5	0.0
O3—Co1—O4—C1	135.93 (6)	C5—N2—C4—O5	180.0
O3^i^—Co1—O4—C1	−135.93 (6)		

Symmetry code: (i) *x*, −*y*+1/2, *z*.

### Hydrogen-bond geometry (Å, º)

**Table:**

*D*—H···*A*	*D*—H	H···*A*	*D*···*A*	*D*—H···*A*
O1—H1*A*···O6^ii^	0.86	1.95	2.792 (4)	171
O1—H1*B*···O6^iii^	0.86	2.26	2.792 (4)	120
C2—H2*D*···O4	0.96	2.34	2.715 (7)	103
O3—H3*A*···O5^iv^	0.85 (2)	1.99 (2)	2.807 (8)	162 (4)
O3—H3*B*···O8^v^	0.88 (2)	1.85 (2)	2.731 (17)	178 (4)
O2—H2*A*···O6^i^	0.88 (2)	1.79 (2)	2.660 (3)	171 (4)
O2—H2*B*···O7^v^	0.89 (4)	1.86 (4)	2.745 (15)	177 (4)

Symmetry codes: (i) *x*, −*y*+1/2, *z*; (ii) −*x*+1/2, −*y*+1, *z*−1/2; (iii) −*x*+1/2, *y*−1/2, *z*−1/2; (iv) *x*−1/2, −*y*+1/2, −*z*+1/2; (v) −*x*+1/2, −*y*, *z*−1/2.
